# Neoadjuvant multidrug chemotherapy including High-Dose Methotrexate modifies VEGF expression in Osteosarcoma: an immunohistochemical analysis

**DOI:** 10.1186/1471-2474-11-34

**Published:** 2010-02-16

**Authors:** Barbara Rossi, Giovanni Schinzari, Giulio Maccauro, Laura Scaramuzzo, Diego Signorelli, Michele A Rosa, Carlo Fabbriciani, Barone Carlo

**Affiliations:** 1 Department of Orthopaedics and Traumatology, Catholic University, Agostino Gemelli Hospital, Rome, Italy; 2 Department of Medical Oncology, Catholic University, Agostino Gemelli Hospital, Rome, Italy; 3 Department of Orthopaedics, Messina University, Messina, Italy

## Abstract

**Background:**

Angiogenesis plays a role in the progression of osteosarcoma, as well as in other mesenchymal tumors and carcinomas, and it is most commonly assessed by vascular endothelial growth factor (VEGF) expression or tumor CD31-positive microvessel density (MVD). Tumor VEGF expression is predictive of poor prognosis, and chemotherapy can affect the selection of angiogenic pattern. The aim of the study was to investigate the clinical and prognostic significance of VEGF and CD31 in osteosarcoma, both at diagnosis and after neoadjuvant chemotherapy, in order to identify a potential role of chemotherapy in angiogenic phenotype.

**Methods:**

A retrospective analysis was performed on 16 patients with high grade osteosarcoma. In each case archival pre-treatment biopsy tissue and post-chemotherapy tumor specimens were immunohistochemically stained against CD31 and VEGF, as markers of angiogenic proliferation both in newly diagnosed primary osteosarcoma and after multidrug chemotherapy including high-dose methotrexate (HDMTX). The correlation between clinicopathological parameters and the degree of tumor VEGF and CD31 expression was statistically assessed using the χ^2 ^test verified with Yates' test for comparison of two groups. Significance was set at *p *< 0,05.

**Results:**

Expression of VEGF was positive in 11 cases/16 of cases at diagnosis. Moreover, 8 cases/16 untreated osteosarcomas were CD31-negative, but the other 8 showed an high expression of CD31. VEGF expression in viable tumor cells after neoadjuvant chemotherapy was observed in all cases; in particular, there was an increased VEGF expression (post-chemotherapy VEGF - biopsy VEGF) in 11 cases/16. CD31 expression increased in 11 cases/16 and decreased in 3 cases after chemotherapy. The data relating to the change in staining following chemotherapy appear statistically significant for VEGF expression (*p *< 0,05), but not for CD31 (*p *> 0,05).

**Conclusions:**

Even if the study included few patients, these results confirm that VEGF and CD31 expression is affected by multidrug chemotherapy including HDMTX. The expression of angiogenic factors that increase microvessel density (MVD) can contribute to the penetration of chemotherapeutic drugs into the tumor in the adjuvant stage of treatment. So VEGF could have a paradoxical effect: it is associated with a poor outcome but it could be a potential target for anti-angiogenic therapy.

## Background

Osteosarcoma is the most common malignant bone tumor in adolescents and young adults [1-3]. Because it is a systemic disease it requires a combined treatment consisting of neoadjuvant chemotherapy, wide tumor excision, adjuvant chemotherapy and, if necessary, resection of metastases. Multimodality treatments have markedly improved the prognosis for patients with osteosarcoma [4,5] and life expectancy is now 10 years for 50-70% of patients [2]. Despite these therapeutic advances and the identification of several prognostic factors [6], pulmonary metastasis occurs in approximately 40-50% of osteosarcoma patients; it is the most frequent cause of death [4,7-11], and there are no effective risk stratification categories. Because it is particularly important to predict the probability of a recurrence of the tumor at an early stage and to customize treatment protocols [7], the possibility of identifying new biological parameters associated with more aggressive tumor behavior and with a poor prognosis could be very useful. Recent studies have focused on the role of angiogenesis in osteosarcoma, albeit with controversial results [8,12,13]. Angiogenesis is known to be a fundamental factor in the local growth of tumors and in progression with metastases, and is most commonly assessed by measuring either the expression of vascular endothelial growth factor (VEGF) in cancer cells or tumor CD31- or CD34-positive microvessel density (MVD). Cancer cells respond to an early hypoxic stage by activating signaling pathways that induce cell proliferation, the production of angiogenic factors such as VEGF and new endothelial cell formation in order to provide a new vascular supply [14,15].

VEGF is a dimeric glycoprotein that is a highly specific mitogen for vascular endothelial cells *in vitro*, as well as inducing migration and preventing apoptosis of these cells *in vivo*; VEGF expression by tumor cells is stimulated by hypoxia, paracrine cytokines and activated oncogenes and it provides a wide surface of permeable CD31-positive microvessels from which tumor cells can be sustained and enter the circulation [4,14,16,17].

VEGF expression in primary tumors and metastases shows a statistically significant correlation with poor prognosis in several pathologies such as breast, lung, renal, gastric, colon-rectal and esophageal carcinomas [18-20]. A correlation between the histological grade of malignancy and VEGF expression has recently been found also in chondrosarcoma[21,22].

Several studies have evaluated the potential role of angiogenesis, and of VEGF in particular, also in osteosarcoma; however the majority of these included heterogeneous series and produced conflicting results because VEGF expression in osteosarcoma was evaluated only before or only after neoadjuvant chemotherapy, in primary tumors and/or in metastases. Nevertheless, these studies demonstrated that VEGF has a predictive significance as a marker of poor prognosis and of the risk of metastasis [4,7,17,23-25]. Recently the prognostic role of post-chemotherapy VEGF expression as well as the changes in VEGF expression following chemotherapy have been evaluated [26,27]: multidrug chemotherapy appeared to reduce VEGF expression by viable tumor cells, even though the series analyzed were not homogeneous in terms of staging or grading and the chemotherapy protocols did not include methotrexate. The rate of necrosis in resected tumor specimens, of more or less than 90% in respectively "good" or "poor" responders to neoadjuvant chemotherapy [3] still remains the more important prognostic factor [1]; however, if chemotherapy can affect tumor angiogenesis, different expression levels of VEGF in osteosarcoma before and after chemotherapy could be considered an additional biologic factor predictive of potential distant metastasis and/or local relapse and a marker of chemosensitivity. The effects of multidrug chemotherapy including high-dose methotrexate (HDMTX) on tumor angiogenesis and VEGF expression are in any case still unknown.

The aim of this study was to investigate the clinical and prognostic significance of VEGF in osteosarcoma and its correlation with CD31-positive microvessel density (MVD), in order to identify a potential role of chemotherapy in an angiogenic phenotype. The Authors report the immunohistochemical results of VEGF expression in 16 cases of osteosarcoma both at diagnosis and after neoadjuvant chemotherapy including HDMTX.

## Methods

A retrospective analysis was performed on 16 patients with high grade osteosarcoma, treated in the Units of Orthopedic Surgery and Clinical Oncology of the Catholic University from 2000 to 2006. For each patient, the clinicopathological characteristics included: age at diagnosis, sex, histological subtype, site, surgical technique, local relapse, metastasis, disease-free and overall survival.

After a detailed history and examination, all patients were subjected to instrumental investigations which included: plain radiographs and CT scan of the affected bone; MRI of the entire affected extremity; plain radiographs and CT scan of the chest to screen patients for metastatic disease. All patients were finally staged according to the Enneking staging system [28]; all received the same treatment consisting of radical tumor excision and multiagent neoadjuvant and adjuvant chemotherapy.

Patients received 2 cycles of preoperative chemotherapy (high dose methotrexate 12000 mg/m^2^/dose, cisplatin 45 mg/m^2^/dose, adriamycin 75 mg/m^2^/dose), according to the Scandinavian osteosarcoma protocol, SSG XIV [10]. Adequate margins were achieved in all cases at the time of surgery.

Four archival tissue blocks were selected from core tumor tissue in each patient; of these, 2 were taken from the diagnostic biopsy and 2 from the resected tumor after neoadjuvant chemotherapy; only viable sections of baseline and resected tumor were evaluated for this study. Samples were immunohistochemically stained for CD31 expression as a specific marker of tumor MVD and for VEGF expression as being indicative of angiogenic proliferation; 5 nm thick serial sections were retrieved from the formalin-fixed paraffin-embedded tissue blocks, deparaffinized in xylene and rehydrated through graded alcohols. Antigen retrieval was performed by microwave heating with 1 mM EDTA (pH 9.0) buffer solution for 12 minutes. Sections were treated with 1% hydrogen peroxide in methanol to block endogenous peroxidase activity. After a brief wash in PBS, 2 sections from each patient (respectively pre and post-treatment) were incubated with purified mouse antihuman CD31 monoclonal antibody at 1:100 dilution (Novocastra); immunolocalization of VEGF was performed using a rabbit polyclonal IgG antibody at 1:100 dilution (Santa Cruz Biotechnologies). Antibody binding was detected with a biotinylated conjugated peroxidase antibody at 1:200 dilution (Vector-Biolab). Immunohistochemical reaction was indirectly amplified using the avidin-biotin-peroxidase complex technique (Vector-Biolab). The specific antibody binding was visualized using 3,3'-diaminobenzidine tetrahydrochloride (DAB, Vector-Biolab). The degree of tumor angiogenesis in terms of microvessel density (MVD) was determined by assessing CD31 as a specific endothelial cell marker. The number of CD31-positive vessels was counted in four randomly selected areas of a 1 mm^2 ^high power field (HPF), and the average was calculated. As a parameter of total surface area of vasculature, the total perimeter of vessels was measured in four randomly selected areas of a 0,25 mm^2 ^field using an image analyzer (Carl Zeiss Axiovision 3.1, Germany); MVD was defined as low (< 10 vessels/HPF), moderate (10-40 vessels/HPF) or high (> 40 vessels/HPF). Assessment of VEGF expression was based on the overall intensity of membranous and cytoplasmic staining within the tumor cells and the number of stained cells/HPF. Four grades were assigned: negative-low (-: < 25 cells/HPF); moderate (+: 25-50 cells/HPF); strong (++: 50-75 cells/HPF) and very strong (+++: > 75 cells/HPF). Negative controls were included by omitting VEGF antibody during the primary antibody incubation, and human thyroid tissue was taken as positive control. Counterstaining was performed with Harris haematoxylin and sections were examined under the light microscope (Carl Zeiss Axioskop 40, Germany) by a single pathologist blinded to the patients' clinical characteristics. No section from resected tumors had 100% post-chemotherapy necrosis, which was evaluated following criteria reported by Picci [3].

### Statistics

Descriptive statistics were calculated. The relationship between clinicopathological parameters and levels of post-chemotherapy tumor VEGF expression was statistically assessed using the χ^2 ^test verified with Yates' test for the comparison of two groups. The relationship between the change in VEGF expression after chemotherapy and disease-free survival and overall survival was analyzed using Kaplan-Meier survival curves. Significance was set at *p *< 0,05. Disease-free survival was evaluated considering the interval between diagnosis and relapse or last follow-up.

## Results

Table 1 summarizes the clinical parameters of 16 patients with high grade osteosarcoma of the extremities treated between 2000 and 2006. The mean age was 17,25 years (range, 13-40 years). Twelve patients were male and 4 were female. At the time of diagnosis, no patient had evidence of metastatic disease. Thirteen tumors were located in the distal femur, 1 in the proximal tibia and 2 in the proximal humerus. The distribution of patients according to the Enneking staging system showed stage IIA in 15 cases (93,75%), and stage IIB in 1 patient (6,95%). All cases were histologically classified as central high grade osteosarcoma, except 1 parosteal osteosarcoma dedifferentiated in central high grade osteosarcoma.

**Table 1:** Patients and clinical characteristics

Patients	16
**Male/Female**	12/4

**Average age** **(range)**	17.25 (13-40)

**Histotype: Central high grade**	15
**Dedifferentiated parosteal**	1

**Site: Distal femur**	13
**Proximal tibia**	1
**Proximal humerus**	2

**Resection: Extra-articular**	1
**Intra-articular**	15

**Reinsertion: Direct**	12
**TREVIRA tube**	4

**Relapse**	5

**Metastasis**	7

**Death**	5

All patients underwent neoadjuvant multiagent chemotherapy including high dose methotrexate [10,29], adriamycin and cisplatin (HDMTX-ADM-CDP) followed by wide surgical resection; limb-sparing procedures included intra-articular (15 cases) and extra-articular (1 case) resection and reconstruction with modular prosthesis (MUTARS, Implantcast^®^).

After evaluation of the degree of necrosis (> or < 90% tumor cells) in the resected tumor specimens following criteria reported by Picci [3], all patients received adjuvant chemotherapy according to current protocols [10,29] and their classification as "good" or "poor" responders: patients classified as good responders were treated for 21 weeks with cycles of ADM, MTX, and CDP, while poor responders received a longer treatment that also included ifosfamide or etoposide, with no severe toxicity-related complications.

Clinical, MRI and chest-XR follow-up was performed every 3 months in the first 2 years, then each six months; the mean follow-up period was 72 months (range, 36-108 months). During the follow-up, 7 patients were affected by lung metastases (43,75%), and 5 also developed local relapse (31,25%). In all cases, relapse was treated with limb amputation. At the time of the latest review (May, 2009) 5 patients (31,25%) affected by both lung metastases and local relapse had died of their disease (Table 1).

**Figure 1:**
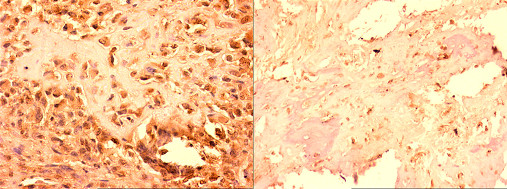
**VEGF expression in osteosarcoma before and after chemotherapy**. Pre-chemotherapy biopsy (×200) and post-chemotherapy resection specimens (×600) showing immunostaining for VEGF.

**Figure 2:**
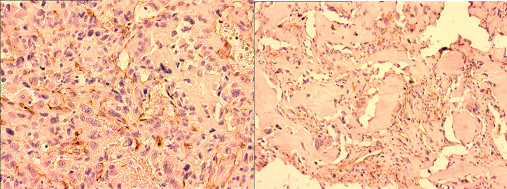
**CD31 expression in osteosarcoma before and after chemotherapy**. Pre-chemotherapy biopsy (×400) and post-chemotherapy resection specimens (×600) showing immunostaining for CD31.

For all 16 primary osteosarcomas, both biopsy and resection specimens were immunohistologically stained using antibodies specific to VEGF and CD31, in order to investigate the clinical significance of angiogenesis in osteosarcoma and the effects of chemotherapy including HDMTX in neo-vascularization (Figures 1, 2).

**Figure 3:**
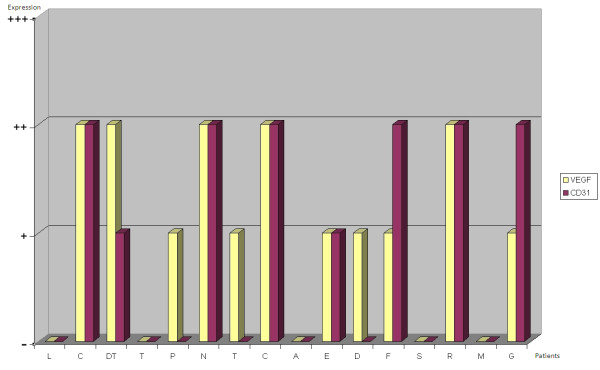
**Bar charts showing levels of VEGF and CD31 expression in pre-chemotherapy biopsy specimens**.

**Figure 4:**
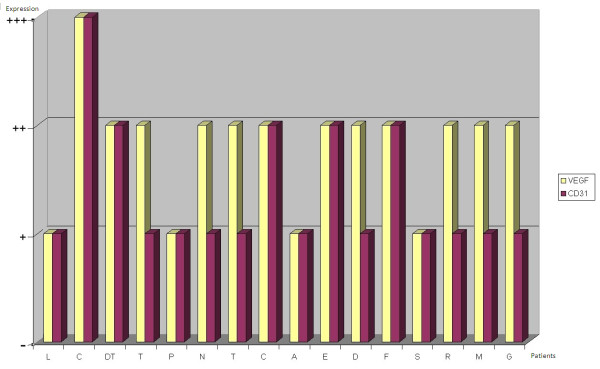
**Bar charts showing levels of VEGF and CD31 expression in post-chemotherapy resection specimens**.

**Figure 5:**
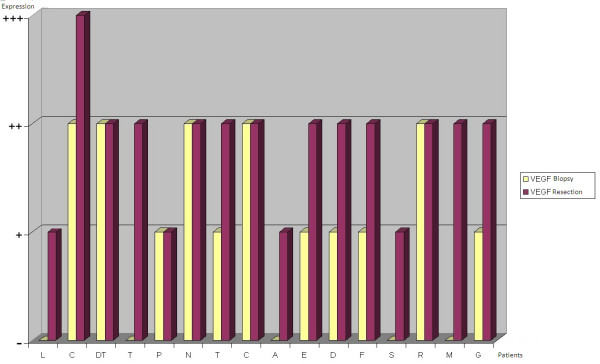
**Changes in VEGF expression after neoadjuvant chemotherapy are compared for each patient**.

**Figure 6:**
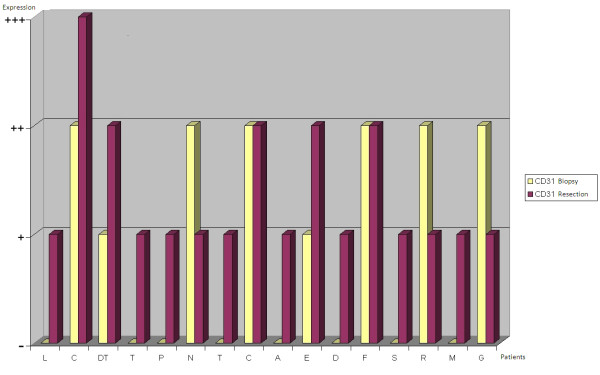
**Changes in CD31 expression after neoadjuvant chemotherapy are compared for each patient**.

**Figure 7:**
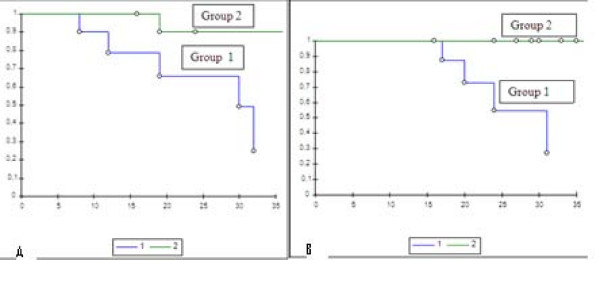
**Kaplan-Meier survival curve in relation to post-chemotherapy VEGF expression in viable tumor cells**. Disease-free (**A**) and overall (**B**) survival of patients affected by osteosarcoma. *Y*-axis = Survival proportion; *X*-axis = follow up in months; group 1 = post-chemotherapy increased VEGF expression; group 2 = post-chemotherapy unchanged VEGF expression.

VEGF staining on biopsy specimens was defined as positive in 11 out of 16 cases (68,75%) (Figure 3): 6 of these (54,5%) showed moderate (+) VEGF staining, while the remaining 5 (45,5%) were strongly (++) VEGF-positive. CD31 was expressed on biopsy specimens in only 50% of cases (Figure 3). At the time of diagnosis VEGF expression was thus more common than CD31 expression. Immunohistochemistry on post-chemotherapy tumor resection specimens showed the expression of VEGF in all 16 treated osteosarcomas, albeit with different degrees of staining in viable tumor cells, and of CD31 in microvessels, even in specimens that were VEGF and/or CD31-negative at biopsy (Figure 4). There was a change in VEGF and CD31 expression following neoadjuvant chemotherapy: in particular, there was an increased VEGF expression in 11 cases/16 and no reduction in VEGF expression was observed after chemotherapy in any case (Figure 5); however, CD31 expression increased in 11 cases/16 and decreased in 3 cases after chemotherapy (Figure 6); in 9 out of 11 cases with over-expression of VEGF (post-chemotherapy VEGF - biopsy VEGF), an increased CD31 staining was also found (Figures 5, 6). The data relating to the change in staining following chemotherapy appear statistically significant for VEGF over-expression (*p *< 0,05), but not for CD31 (*p *> 0,05). No association was found between post-chemotherapy VEGF expression and patient gender or age (Table 2), but a correlation with anatomical localization was observed (*p *< 0,05). Furthermore, a statistically significant association was found between VEGF over-expression in treated osteosarcomas and local relapse, lung metastasis and survival (Table 2). The highest VEGF-positive (++, +++) osteosarcomas at biopsy with increased VEGF expression after treatment were the most clinically aggressive tumors; they developed local relapse and/or lung metastases earlier and led to the death of patients (*p *< 0,05). Kaplan-Meier survival curves (Figure 7A, B) showed a poorer prognosis for patients who had an increased VEGF expression after chemotherapy than for those whose post-treatment VEGF expression was unchanged.

**Table 2:** Correlation between unchanged (↔) and increased (↑) post-chemotherapy VEGF expression and clinicopathological parameters; statistics significance p < 0,05 with 1 grade of freedom.

Parameters	VEGF immunostaining after CHT		*p*
	VEGF ↔	VEGF ↑	
**Age**			
≥ 17	1	7	
< 17	4	4	*p *= 0.11

**Sex**			
Male	4	8	
Female	1	3	*p *= 0.11

**Site**			
Femur	2	11	
Other bones	3	0	*p *= 0.02

**Relapse**			
Present	0	5	
Absent	5	6	*p *= 0.04

**Metastases**			
Present	1	6	
Absent	4	5	*p *= 0.04

**Survival**			
Dead	0	5	
Alive	5	6	*p *= 0.04

## Discussion

Osteosarcoma is a systemic disease and hematogenous spreading is therefore essential for its local proliferation and dissemination to distant parts of the anatomy [1].

It is well-known that angiogenesis is an early stage in the growth of carcinomas and mesenchymal tumors: VEGF expression correlates with stage, grade and prognosis of patients with gastro-enteric, lung and breast carcinomas, soft-tissue sarcomas and chondrosarcomas [12,18-20,22].

Several studies have examined the clinical significance of angiogenesis-related biomarkers (VEGF, CD31, CD34, *etc*.) in osteosarcoma, but their results are at times in conflict and few reports relate to studies with large series.

According to Kaya [4] VEGF-positive immunostaining in untreated tumor specimens was predictive of lung metastases and of short overall and disease-free survival. This finding was also assessed by Charity [17] in a retrospective study on resected stage IIB osteosarcomas around the knee: VEGF expression in more than 25% of tumor cells after neoadjuvant chemotherapy correlated with shorter overall and disease-free survival. In contrast, in a larger series analyzed by Kreuter [8], the measurement of high levels of CD31-positive microvessel density in pretreatment tumor samples was a favorable prognostic factor and it was associated with a good response to chemotherapy.

Ek [13] evaluated the expression of VEGF and CD31/34 as markers of MVD in pre-treatment biopsy and found that all were expressed moderately or strongly in almost all specimens, showing that osteosarcoma is marked by moderate-to-high tumor microvessel density. However, there was no statistical correlation between VEGF expression, MVD, clinicopathological features and disease outcomes.

A recent immunohistochemical study focused on hypoxia-inducible factor (HIF-1α) and VEGF expression as markers of hypoxia in specimens from primary and metastatic sites: a higher level of VEGF and HIF-1α expression was observed in pulmonary metastases, showing a potential correlation with prognosis and response to chemotherapy. No correlation between VEGF, micro-vascular density and clinical outcomes was found in this study either [14]. None of these studies considered the possible role of chemotherapy in the selection of angiogenic patterns in osteosarcoma.

VEGF and αV integrin expression were compared for the first time in untreated osteosarcomas with different Enneking stages and in the same patients after chemotherapy (ADM-CDP-IFO) by Huang [26], confirming VEGF expression in osteosarcoma before treatment. Interestingly, the strong VEGF and αV integrin staining were significantly reduced after chemotherapy; however, residual strong staining of VEGF but not of αV integrin in resected tumors was associated with advanced Enneking stages and a higher incidence of local relapse and distant metastases. This study did not take into account the role of HDMTX-inclusive chemotherapy in osteosarcoma.

Bajpai [27] analyzed both baseline and post-chemotherapy VEGF expression in a series of 31 patients affected by osteosarcoma, finding 90% agreement between a positive baseline VEGF expression and a higher histological grade; moreover, the decrease in VEGF expression in the viable cells following chemotherapy showed a significant association with favourable histological necrosis. Thus, high VEGF expression in tumor cells surviving in post-chemotherapy resected osteosarcomas is an important negative prognostic factor, because it is related to poor chemosensitivity. However, this series included patients with a disproportionate stage distribution, both metastatic and non-metastatic at diagnosis, and treated with only three cycles of cisplatin and doxorubicin.

In the present series, VEGF expression was evaluated through immunohistochemical analysis both at diagnostic biopsy and in the resected tumor specimens of 16 patients affected by non-metastatic high grade osteosarcoma of the extremities, in order to define a potential effect of neoadjuvant chemotherapy with HDMTX on tumor angiogenesis. Interestingly, patients included in this study belonged to a homogeneous series in terms of grade of malignancy and Enneking staging (IIA in 15 cases/16); the only patient affected by a parosteal osteosarcoma (stage IIB) had the same prognosis as the other patients because of the histological dedifferentiation in central high grade osteosarcoma [28]. Moreover, the Authors attempted to identify a correlation between the change in angiogenic pattern following chemotherapy, the tendency to local relapse and/or metastases and survival.

In accordance with the literature the study demonstrates that a moderate-high microvessel density (MVD) is a typical aspect of osteosarcoma at the time of presentation, as shown by the moderate-to-strong VEGF and CD31 expression found respectively in about 70% and 50% of biopsy specimens. VEGF expression is higher than CD31 at biopsy, suggesting that VEGF is a more objective and sensitive marker of angiogenic regulation; it can therefore be used to evaluate the prognostic role of angiogenesis in osteosarcoma, as well as in other carcinomas and mesenchymal tumors [12,18,19,22,25,26]. The comparison of VEGF and CD31 expression in biopsy and resection specimens suggests the possibility that neoadjuvant chemotherapy might influence angiogenesis in osteosarcoma. Preoperative chemotherapy including HDMTX seems to increase angiogenesis in high grade osteosarcoma, because VEGF and CD31 are over-expressed (post-chemotherapy VEGF - biopsy VEGF) in the majority of resected tumor specimens. Tumor necrosis and/or chemo-induced hypoxia could promote the up-regulation of VEGF by viable tumor cells and then the proliferation of CD31-positive endothelial cells. There was an increase in VEGF and CD31 expression after multidrug chemotherapy including HDMTX, but this was statistically significant only for VEGF (*p *< 0,05) probably because too few patients were included in the series. Epidemiologic parameters such as patients' gender and age were not significantly associated with VEGF over-expression after chemotherapy; on the contrary, a correlation with femoral localization was found (*p *< 0,05), mainly due to the disproportionate distribution of cases, as 13 cases out of 16 were localized in the femur. In the present series, 5 patients out of 16 (31,25%) developed both local relapse and pulmonary metastases and subsequently died. Interestingly, the increase of VEGF expression after preoperative chemotherapy in these patients positively correlated with relapse of osteosarcoma, lung metastases and survival (*p *< 0,05).

The Authors assume that different results between this study and Hoang' [26] or Bapjai's [27] reports could be related to different multidrug protocols (MTX vs ifosfamide), but they cannot conclude that MTX is the primary cause of that. Further study should be performed in order to conclude about it.

Angiogenesis in osteosarcoma can be considered as a diagnostic marker of tumor progression, in terms of local growth and metastatic potential: in this series, the change in VEGF following chemotherapy correlates with a poorer prognosis. Furthermore, increased VEGF expression by viable tumor cells in resected tumors should be considered as a prognostic marker of a more aggressive phenotype after neoadjuvant chemotherapy, because chemo-resistant tumor cells are potentially responsible for local relapse and/or metastases. Together with low (< 90%) tumor necrosis [1,3], the increase in VEGF expression after neoadjuvant chemotherapy could be an additional biological marker predictive of a poor response to treatment and poor prognosis. On the other hand, as a consequence of the increase in angiogenic factors, high microvessel density (MVD) could facilitate the spreading of chemotherapeutic drugs in the adjuvant stage of treatment.

The effects of anti-angiogenic therapy in the treatment of osteosarcoma have been investigated in mouse tumor models [5,15], but not yet in clinical trials. However, Tsunemi [5] and Kaya [30] found concomitant tumor resistance both in animal models and in 10 patients with osteosarcoma: the removal of the primary tumor significantly promotes the early development of distant metastases through the activation of systemic angiogenesis. Thus, patients with osteosarcoma in whom serum levels of VEGF are post-operatively elevated could be good candidates for adjuvant anti-angiogenic therapy in order to prevent progression of distant metastases.

In conclusion, VEGF could have a role as a marker predictive of poor prognosis after neoadjuvant chemotherapy, but also as a specific target for post-operative anti-angiogenic therapy. Thus, anti-angiogenic therapy could augment the effects of adjuvant chemotherapy through the inhibition of VEGF, especially in elderly patients, in spinal or pelvic sites that are difficult to treat with wide surgery [6], and most of all, in "poor responders" to neoadjuvant chemotherapy, who are usually affected by the highest VEGF-positive osteosarcomas.

## Conclusions

Although multidrug chemotherapy and wide surgery have markedly improved the prognosis of osteosarcoma, it is necessary to find some new molecular targets and therapeutic solutions in order to deal with pulmonary metastases and local relapse, which are still a real challenge in the treatment of this tumor. Several studies have focused on the role of angiogenesis in osteosarcoma and research is being directed towards anti-angiogenetic factors in order to reduce its metastatic potential. In this study chemotherapy-induced VEGF expression in resected tumor specimens is shown to be a negative prognostic factor correlated with local and systemic progression; nevertheless, VEGF expression after neoadjuvant chemotherapy should be considered a specific target for adjuvant anti-angiogenic therapy.

## Competing interests

The authors declare that they have no competing interests.

## Authors' contributions

GM and MAR are the surgeons who operated on patients. CB and GS followed patients during chemotherapy. BR drafted the manuscript. GS, BR and DS performed immunohistochemical assays. LS performed the statistical analysis. GM and GS conceived the study, participated in its design and coordination and helped to draft the manuscript. CF coordinated the group. All the authors have read and approved the final manuscript.

## Pre-publication history

The pre-publication history for this paper can be accessed here:

http://www.biomedcentral.com/1471-2474/11/34/prepub

## References

[B1] Bacci G Ferrari S Bertoni F Picci P Bacchini P Longhi A Donati D Forni C Campanacci L Campanacci M Histologic response of high-grade nonmetastatic osteosarcoma of the extremity to chemotherapy CORR 2001 386 186 196 10.1097/00003086-200105000-00024 11347833

[B2] Meyers PA Pappo A Osteosarcoma Pediatric Bone and Soft Tissue Sarcomas 2006 1 Springer 219 228 full_text

[B3] Picci P Bacci G Campanacci M Gasparini M Pilotti S Cerasoli S Bertoni F Guerra A Capanna R Albisinni U Galletti S Gherlinzoni F Calderoni P Sudanese A Baldini N Bernini M Jaffe N Histological evaluation of necrosis in osteosarcoma induced by chemotherapy. Regional mapping of viable and non viable tumour Cancer 1985 56 1515 21 10.1002/1097-0142(19851001)56:7<1515::AID-CNCR2820560707>3.0.CO;2-6 3861228

[B4] Kaya M Wada T Akatsuka T Kawaguchi S Nagoya S Shindoh M Higashino F Mezawa F Okada F Ishii S Vascular endothelial growth factor expression in untreated osteosarcoma is predictive of pulmonary metastasis and poor prognosis Clin Cancer Res 2000 6 2 572 77 10690541

[B5] Tsunemi T Nagoya S Kaya M Kawaguchi S Wada T Yamashita T Ishii S Postoperative progression of pulmonary metastasis in osteosarcoma CORR 2003 407 159 66 10.1097/00003086-200302000-00024 12567143

[B6] Bielack SS Kempf-Bielack B Delling G Exner GU Flege S Helmke K Kotz R Salzer-Kuntschik M Werner M Winkelmann W Zoubek A Jurgens H Winkler K Prognostic factors in high-grade osteosarcoma of the extremities or trunk: an analysis of 1.702 patients treated on neoadjuvant Cooperative Osteosarcoma Study Group Protocols J Clin Oncol 2002 20 776 790 10.1200/JCO.20.3.776 11821461

[B7] Kaya M Wada T Kawaguchi S Nagoya S Yamashita T Abe Y Hiraga H Isu K Shindoh M Higashino F Okada F Tada M Yamawaki S Ishii S Increased pre-therapeutic serum vascular endothelial growth factor in patients with early clinical relapse of osteosarcoma Br J Cancer 2002 86 864 9 10.1038/sj.bjc.6600201 11953816 PMC2364146

[B8] Kreuter M Bieker R Bielack SS Auras T Buerger H Gosheger G Jurgens H Berdel WE Mesters RM Prognostic relevance of increased angiogenesis in osteosarcoma Clin Canc Res 2004 10 8531 7 10.1158/1078-0432.CCR-04-0969 15623635

[B9] Link MP Goorin MA Miser AW Green AA Pratt CB Belasco JB Pritchard J Malpas JS Baker AR Kirkpatrick JA The effect of neoadjuvant chemotherapy on relapse-free survival in patients with osteosarcoma of the extremity N Engl J Med 1986 314 1600 6 3520317 10.1056/NEJM198606193142502

[B10] Samardziski M Zafiroski G Tolevska C Zafirova-Ivanovska B Kostadinova-Kunovska S Kalikanin-Markovska M Treatment of high-grade non-metastatic osteosarcoma (study of 30 cases treated with Scandinavian osteosarcoma protocol XIV and surgery) Prilozi 2009 29 2 309 24 19259055

[B11] Wada T Isu K Takeda N Usui M Ishii S Yamawaki S A preliminary report of neoadjuvant chemotherapy NSH-7 study in osteosarcoma: preoperative salvage chemotherapy based on clinical tumour response and the use of granulocyte colony-stimulating factor Oncology 1996 53 221 7 10.1159/000227564 8643225

[B12] DuBois S Demetri G Markers of angiogenesis and clinical features in patients with sarcoma Cancer 2007 109 5 813 9 Review. 10.1002/cncr.22455 17265525

[B13] Ek ET Ojaimi J Kitagawa Y Choong PF Outcome of patients with osteosarcoma over 40 years of age: is angiogenesis a marker of survival? Int Semin Surg Oncol 2006 21 3 7 10.1186/1477-7800-3-7 PMC1435760 16551370

[B14] Mizobuchi H Garcìa-Castellano JM Philips S Healey JH Gorlick R Hypoxia markers in human osteosarcoma: an exploratory study CORR 2008 446 9 2052 9 10.1007/s11999-008-0328-y PMC2493019 18528739

[B15] Rennel E Waine E Guan H Schuler Y Leenders W Woolard J Sugiono M Gillatt D Kleinerman E Bates D Harper S The endogenous anti-angiogenetic VEGF isoform, VEGF165b inhibits human tumour growth in mice Br J Cancer 2008 98 7 1250 7 10.1038/sj.bjc.6604309 18349828 PMC2359649

[B16] Bergers G Benjamin LE Tumorigenesis and the angiogenic switch Nat Rev Cancer 2003 3 6 401 10 Review. 10.1038/nrc1093 12778130

[B17] Charity RM Foukas AF Deshmukh NS Grimer RJ Vascular endothelial growth factor expression in osteosarcoma CORR 2006 448 193 8 10.1097/01.blo.0000205877.05093.c9 16826116

[B18] Inoue K Ozeki Y Suganuma T Sugiura Y Takana S Vascular endothelial growth factor expression in primary esophageal squamous cell carcinoma Cancer 1997 79 206 13 10.1002/(SICI)1097-0142(19970115)79:2<206::AID-CNCR2>3.0.CO;2-I 9010092

[B19] Ishigami SI Arii S Niwano M Harada T Mizumoto M Mori A Onodera H Imamura M Predictive value of vascular endothelial growth factor (VEGF) in metastasis and prognosis of human colorectal cancer Br J Cancer 1998 78 1379 84 9823983 10.1038/bjc.1998.688 PMC2063176

[B20] Maeda H Chung Y Ogawa Y Takatsuka S Kang SM Ogawa M Sawada T Sowa M Prognostic value of vascular endothelial growth factor expression in gastric carcinoma Cancer 1996 77 858 63 10.1002/(SICI)1097-0142(19960301)77:5<858::AID-CNCR8>3.0.CO;2-A 8608475

[B21] Ayala G Liu C Nicosia R Horowitz S Lackman R Microvasculature and VEGF expression in cartilaginous tumors Hum Pathol 2000 31 341 6 10.1016/S0046-8177(00)80248-8 10746677

[B22] Falcone G Rossi ED Maccauro G de Santis V Rosa MA Capelli A Fadda G Diagnostic relevance of the immunohistochemical detection of growth factors in benign and malignant cartilaginous tumors Appl Immunohistochem Mol Morphol 2006 14 3 334 40 10.1097/00129039-200609000-00013 16932026

[B23] Lee YH Tokunaga T Oshika Y Suto R Yanagisawa K Tomisawa M Fukuda H Nakano H Abe S Tateishi A Kijima H Yamazaki H Tamaoki N Ueyama Y Nakamura M Cell-retained isoforms of vascular endothelial growth factor (VEGF) are correlated with poor prognosis in osteosarcoma Eur J Cancer 1999 35 7 1089 93 10.1016/S0959-8049(99)00073-8 10533453

[B24] Oda Y Yamamoto H Tamiya S Matsuda S Tanaka K Yokoyama R Iwamoto Y Tsuneyoshi M CXCR4 and VEGF expression in the primary site and in metastatic site of human osteosarcoma: analysis within a group of patients, all of whom developed lung metastasis Mod Pathol 2006 19 5 738 45 10.1038/modpathol.3800587 16528367

[B25] Kaya M Wada T Nagoya S Sasaki M Matsumura T Yamashita T The level of vascular endothelial factor as a predictor of a poor prognosis in osteosarcoma J Bone Joint Surg Br 2009 91 6 784 8 10.1302/0301-620X.91B6.21853 19483233

[B26] Huang Y Lin Z Zhuang J Chen Y Lin J Prognostic significance of alpha V integrin and VEGF in osteosarcoma after chemotherapy Onkologie 2008 31 10 535 40 10.1159/000151685 18854653

[B27] Bajpai J Sharma M Sreenivas V Kumar R Gamnagatti S Khan SA Rastogi S Malhotra A Bakhshi S VEGF expression as a prognostic marker in osteosarcoma Pediatr Blood Cancer 2009 53 6 1035 9 10.1002/pbc.22178 19621435

[B28] Enneking WF A system of staging musculoskeletal neoplasms Clin Orthop Relat Res 1986 204 9 24 3456859

[B29] Whelan J Seddon B Perisoglou M Management of osteosarcoma Curr Treat Options Oncol 2006 7 6 444 55 Review. 10.1007/s11864-006-0020-y 17032557

[B30] Kaya M Wada T Nagoya S Kawaguchi S Isu K Yamashita T Concomitant tumour resistance in patients with osteosarcoma. A clue to a new therapeutic strategy J Bone Joint Surg Br 2004 86 1 143 7 14765882

